# Association of Adherence to Endocrine Therapy Among Patients With Breast Cancer and Potential Drug-Drug Interactions

**DOI:** 10.1001/jamanetworkopen.2022.44849

**Published:** 2022-12-02

**Authors:** Elie Rassy, Aurélie Bardet, Omar Bougacha, Laurène Gantzer, Béranger Lekens, Suzette Delaloge, Fabrice André, Stefan Michiels, Barbara Pistilli

**Affiliations:** 1 Department of Medical Oncology, Gustave Roussy, University Paris-Saclay, Villejuif, France; 2 Department of Biostatistics and Epidemiology, Gustave Roussy, University Paris-Saclay, Villejuif, France; 3 Oncostat U1018, Inserm, University Paris-Saclay, Ligue Contre le Cancer, Villejuif, France; 4 Research and Development, Cegedim Healthcare Solutions, Boulogne-Billancourt, Paris, France

## Abstract

**Importance:**

Suboptimal adherence to endocrine therapy (ET) among patients with hormone-receptor–positive breast cancer significantly affects survival outcomes and is associated with higher hospitalization rates and health care costs. Weak adherence to long-term treatments has multiple determinants, including disease characteristics, treatment adverse effects, and patients’ attributes, such as age and comorbidities.

**Objective:**

To examine whether potential drug-drug interactions (PDDI) with tamoxifen or aromatase inhibitor were associated with adherence to ET in patients with early and advanced breast cancer.

**Design, Setting, and Participants:**

This cohort study used anonymized health record data of women with breast cancer who received ET in a private observational primary care database. Patients eligible for analysis included women aged 18 years or older who had a reported diagnosis of breast cancer and received ET with tamoxifen or aromatase inhibitor between 1994 and 2021. Data were analyzed 2021.

**Exposures:**

Adherence to ET during a given year was defined by a medication possession ratio of 80% or greater over 1-year prescription periods. PDDI were categorized into absent, minor (a combination to take into account), moderate (combination requiring precautions for use), major (combination not recommended), and contraindicated according to guidelines in the Claude Bernard Drug Database.

**Main Outcomes and Measures:**

We used regression models to estimate odds ratios (ORs) and 95% CIs for the associations between adherence and age, baseline comorbidities, PDDI, and adherence to ET during the previous year.

**Results:**

A total of 10 863 patients who were prescribed ET for breast cancer were eligible for the analysis (age 70 years or older, 3509 patients [32.3%]). In the tamoxifen cohort (3564 patients), PDDI were reported in 497 of 3670 patients (13.5%) at baseline (moderate, 254 patients [51.1%]; major, 227 patients [45.7%]), 2047 of 4831 patients (42.4%) at year 1, 1127 of 2751 patients (41.0%) at year 2, 761 of 1861 patients (40.9%) at year 3, 376 of 1058 patients (35.5%) at year 4, and 201 of 593 patients (33.9%) at year 5. In the aromatase inhibitor cohort (7299 patients), PDDI were reported in 592 of 7437 patients (8.0%) at baseline (moderate in 588 of 592 patients [99.3%]), which reached 2875 of 9031 patients (31.8%) at year 1 and ranged between 31.4% (1802 of 5730 patients in year 2) and 32.8% (791 of 2411 in year 4) throughout the study period. No association between adherence and PDDI was found in the tamoxifen (OR, 0.99; 95% CI, 0.91-1.08) or aromatase inhibitor (OR, 1.05; 95% CI, 0.95-1.15) cohort.

**Conclusions and Relevance:**

In this cohort of patients with hormone-receptor–positive breast cancer, PDDI with tamoxifen and aromatase inhibitors were not associated with adherence to ET.

## Key Points

**Question:**

Are potential drug-drug interactions (PDDI) associated with adherence to endocrine therapy among patients with breast cancer?

**Findings:**

In this cohort study of 10 863 patients who were prescribed endocrine therapy for breast cancer, PDDI were not associated with the adherence to endocrine therapy in the tamoxifen or aromatase inhibitor cohort. No statistically significant differences were observed between the different PDDI categories compared with no PDDI.

**Meaning:**

While these results suggest that PDDI are not associated with adherence to endocrine therapy over time, the considerable proportion of patients with PDDI warrants comprehensive medication assessment at each patient visit to avoid deleterious interactions.

## Teaser

This cohort study of patients prescribed endocrine therapy for breast cancer examines the association of potential drug-drug interactions with therapy adherence.

## Introduction

Endocrine therapy (ET), which includes tamoxifen and aromatase inhibitors (AI) either alone or in combination with other agents, represents the backbone of therapeutic strategies in the management of patients with hormone receptor (HR)-positive breast cancer.^1,2,3^ Multiple large studies have demonstrated associations of ET with improved survival outcomes among these patients. For instance, the meta-analyses performed by the Early Breast Cancer Trialists’ Collaborative Group^4^ evaluating individual patient-level data from multiple clinical trials have shown that adjuvant ET almost halved the recurrence rates and reduced by approximately one-third the mortality rates. In the metastatic setting, ET alone or in combination with targeted agents reduced tumor burden and prolonged survival.^5^

Given that the survival of patients with HR-positive breast cancer is strongly correlated with the use of ET, especially in the adjuvant setting, nonadherence represents a major concern in these patients. In our experience within the French CANTO cohort (NCT01993498), patients who had lower levels of serum tamoxifen were at higher risk of developing distant recurrences early after treatment discontinuation/interruption.^6^ The weak adherence to ET has been associated with multiple determinants, including extremes of age,^7,8^ low socioeconomic status,^9,10,11^ limited information about the benefit of adjuvant ET,^12,13^ and intolerable adverse events, mainly menopausal symptoms.^14,15,16^ Polypharmacy and drug interactions are often overlooked even though they can be encountered in 50% to 91% of patients with breast cancer and can be associated with medication nonadherence, and with mortality among patients with cancer.^17,18,19^ It has also been reported that specific medication classes were associated with adherence to ET among patients with breast cancer.^20^ Although in this case, the patient’s personal attitude to adherence and the mitigation of ET adverse effects by comedications may play a key role in adherence to ET, potential drug-drug interactions (PDDI) may cause significant morbidity and compromise adherence by enhancing drug toxicity.^21,22^ Thus, a comprehensive understanding of drug interactions is central to optimizing patient adherence to ET through the implementation of personalized strategies. This study aimed to explore the PDDI between comedications and ET and investigate their association with adherence to ET.

## Methods

We obtained data on patient health records from the French version of The Health Improvement Network (THIN), a private database that is enriched regularly with patient data from general practitioners using electronic health record software (Cegedim Healthcare). Medical records collected at the physician level of around 2000 general practitioners were coded according to the *International Statistical Classification of Diseases and Related Health Problems, Tenth Revision *(*ICD-10*) codes.

The THIN database obtained approval from the French National Data Protection Authority for data collection in 2002. It comprised fully anonymized electronic medical records compliant with the European general data protection regulations. As the study was a retrospective analysis using secondary anonymized patient data only without reporting on personal information, the French legislation did not require additional ethical approval and subsequently the requirement for obtaining informed consent was waived. This study followed the Strengthening the Reporting of Observational Studies in Epidemiology (STROBE) reporting guideline.

### Patient Selection

For the purpose of this study, we included all women aged 18 years or older who had a reported diagnosis of breast cancer (*ICD-10* code C50.x) and have completed or are undergoing ET with either tamoxifen (Anatomical Therapeutic Chemical [ATC] class L02BA01) or AI (ATC classes L02BG.x; letrozole, anastrozole, exemestane) between 1994 and 2021. Eligible patients had to have at least 1 year of inclusion in the database and have consulted the general practitioner before and after the initiation of ET to ensure the completeness of the data. Data on patient race or ethnicity were not available in the THIN database.

### Variable Assessment

#### Definition of Adherence to Endocrine Therapy

We retrieved the prescription and reimbursement data of the ET to compute the medication possession ratio (MPR). MPR is defined by the proportion of a time period where a medication supply is available. In a given 1-year period, MPR is calculated by dividing the duration of ET prescribed by the duration between 2 consecutive dispenses (eMethods in the Supplement). Discontinuation of ET was determined by having zero dispensing in the following interval or having an observed gap of 30 or more days between the end of the previous supply and the subsequent dispensing of ET. An MPR of 0.80 or higher defined adherence to therapy among patients who received at least 1 ET prescription and included nonadherent patients during the previous interval or switchers to other ET.^23^

#### Definition of PDDI

Comedications at baseline and during subsequent years of treatment with ET were retrieved from the pharmacy dispensing data. Drugs were classified according to the ATC classification system. For every 1-year interval, we collected data on comedications and data on the dispensing of specific medication classes: lipid-lowering agents, antihypertensives, oral diabetes medications, insulin analogs, antidepressants, anxiolytics and antipsychotics, and opioid-containing analgesics. The medication use for each of the classes of interest during 12-month intervals was defined according to nonuse (no dispensing), infrequent use (1 or 2 dispensings), and frequent use (3 or more dispensings). The exposure to these medication classes was limited to the year of the prescription and did not carry forward to subsequent years of ET unless the prescription was renewed. PDDI between daily medication and ET were analyzed using the Claude Bernard Drug Database and were categorized into absent, minor (a combination to take into account), moderate (combination requiring precautions for use), major (combination not recommended), and contraindicated, accordingly.^24^ Given that the adverse events related to ET were not collected in the database, we evaluated the consumption of drugs that are commonly prescribed to manage usual adverse events of ET, including nonsteroidal anti-inflammatory drugs, paracetamol-based combinations, duloxetine, venlafaxine, and oxybutynin. The baseline prescriptions were defined by the prescription of the aforementioned drugs within the year before the first prescription of ET.

### Statistical Analysis

The tamoxifen and AI cohorts were analyzed separately given the potential differences in the ET indications according to patient characteristics; patients switching ET were analyzed within their initial ET cohort. Patients were described using frequencies and proportion for categorical variables. For the adherence parameter, MPR, we used repeated-measures regression models to estimate odds ratios (ORs) and 95% CIs to account for the correlation across time periods of observations contributed by each patient. The multivariable analysis was adjusted for age, baseline comorbidities, PDDI (the worst interaction was retained for the model), and adherence during the previous year. We performed 2 sensitivity analyses taking into consideration comedication instead of baseline comorbidities, with and without PDDI, respectively. All tests were 2-sided and a *P* value <.05 was considered statistically significant. All statistical analyses were conducted using with the SciPy 1.6.3 and Pandas 1.2.1 libraries in Python version 3.9.2.^25^

## Results

### Patient Characteristics

**Figure 1.**
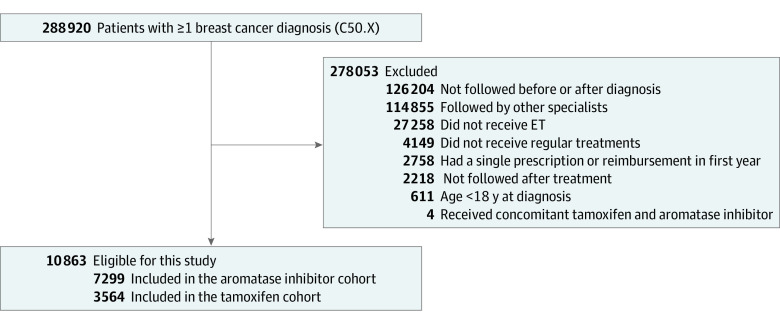
Participant Flow Diagram ET indicates endocrine therapy.

The database included 47 250 patients with breast cancer, among whom 19 992 patients received ET during their cancer care. After selecting those with a minimum follow-up of 1 year, 10 863 patients were included in this analysis (Figure 1). The majority of participants were aged 50 years and above (9131 [84.1%]; age 70 years or older, 3509 patients [32.3%]) and a minority were younger than 30 years of age (14 [0.1%]) (Table 1). The number of patients with coprescribed medications during years 1 to 5 is reported in eFigure 1 and the frequency of comedication use during ET in eTable 1 in the Supplement.

**Table 1.** Characteristics of the Patients With Hormone Receptor–Positive Breast Cancer Undergoing Endocrine Therapy

Characteristics	Patients, No. (%)	
	Tamoxifen (n = 3564)	Aromatase inhibitor (n = 7299)
Age categories, y		
<30	11 (0.3)	3 (<0.1)
30-39	234 (6.6)	23 (0.3)
40-49	1264 (35.5)	196 (2.7)
50-59	1133 (31.8)	1480 (20.3)
60-69	408 (11.4)	2601 (35.6)
≥70	514 (14.4)	2995 (41.0)
Comorbidities		
Coronary artery disease	12 (0.3)	53 (0.7)
Hypertension	950 (26.7)	3708 (50.8)
Diabetes	193 (5.4)	1008 (13.8)
Thyroid dysfunction	16 (0.4)	42 (0.6)
Rheumatologic disease	57 (1.6)	225 (3.1)
Osteoarthritis	454 (12.7)	1883 (25.8)
Epilepsy	35 (1.0)	98 (1.3)
Dementia	5 (0.1)	18 (0.2)
Cerebrovascular disease	112 (3.1)	393 (5.4)
COPD	46 (1.3)	190 (2.6)
Asthma	370 (10.4)	916 (12.5)
Depression	1076 (30.2)	2264 (31.0)
Switching therapy	1085 (30.4)	656 (9.0)
No. of prescribed drugs during endocrine therapy at baseline^a^		
None	162 (4.5)	139 (1.9)
1-4	622 (17.5)	926 (12.7)
5-9	876 (24.6)	1820 (24.9)
10-15	818 (23.0)	1966 (26.9)
>15	1086 (30.5)	2448 (33.5)

Abbreviation: COPD, chronic obstructive pulmonary disorder.

^a^ Within 1 year before first prescription of endocrine therapy.

### Potential Drug-Drug Interactions

**Figure 2.**
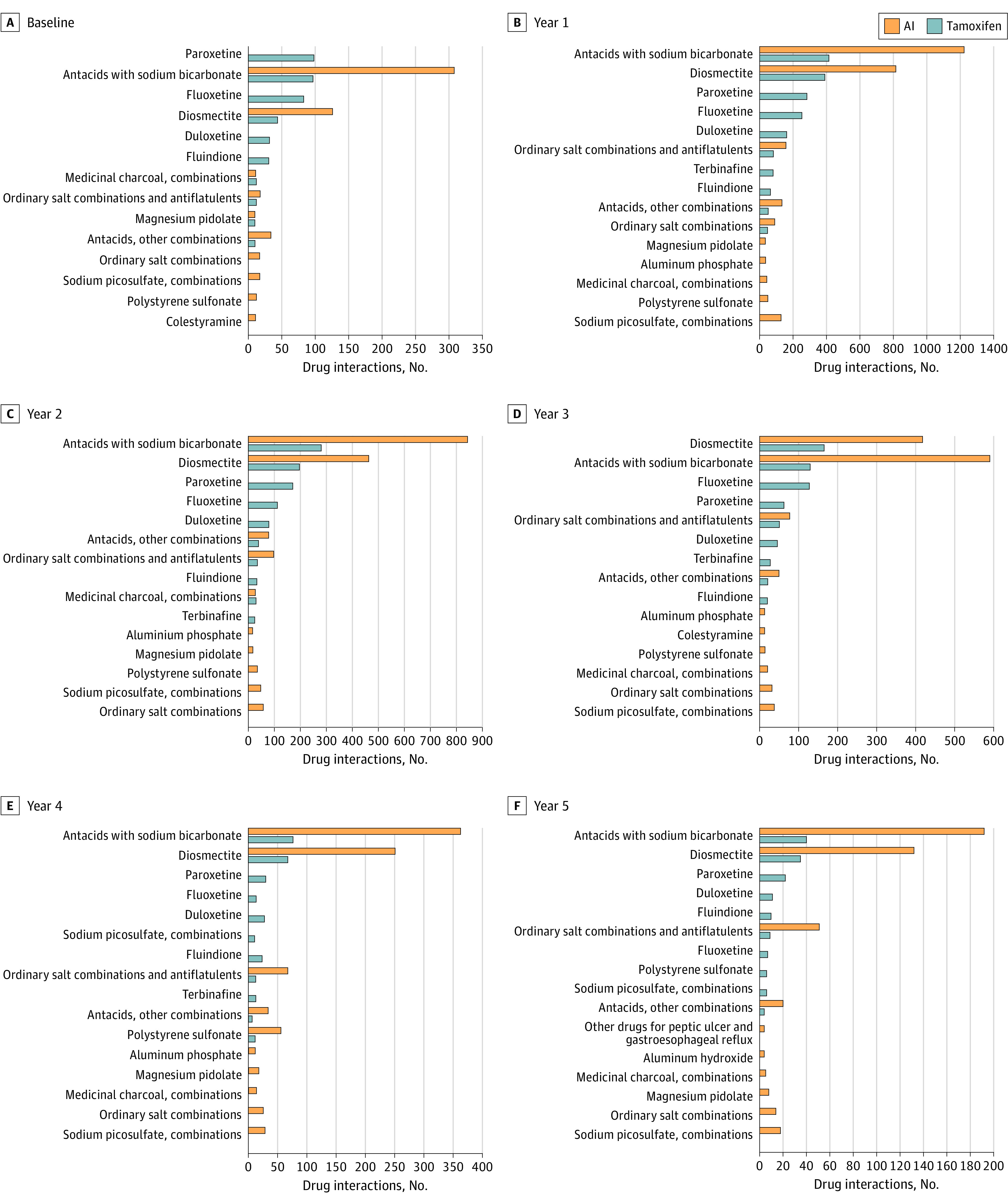
Ranking of the Most Prevalent Drugs Causing Potential Drug-Drug Interaction With Endocrine Therapy

**Figure 3.**
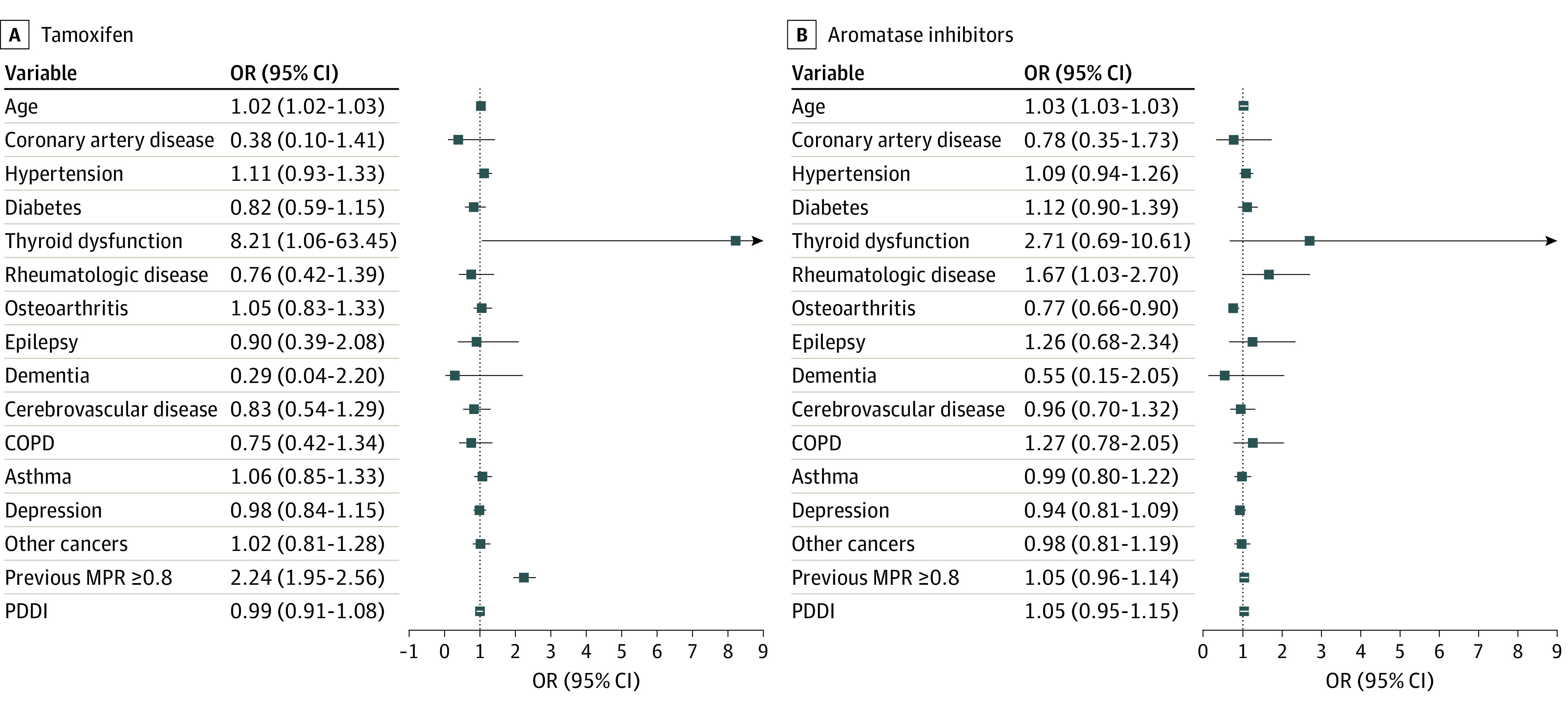
Association of Medication Possession Ratio (MPR) With Baseline Features in the Tamoxifen and Aromatase Inhibitors Cohorts COPD indicates chronic obstructive pulmonary disorder; OR, odds ratio; PDDI, potential drug-drug interactions.

At baseline, PDDI were identified in 391 patients (11.0%) of the tamoxifen cohort and 454 patients (5.7%) in the AI cohort. In the tamoxifen cohort, 497 of 3670 patients (13.5%) had prescriptions that had PDDI at baseline (Figure 2). Most of the PDDI were moderate (254 of 497 patients [51.1%]) and major (227 of 497 patients [45.7%]), a small proportion being minor (14 of 497 patients [2.8%]) or contraindicated (2 of 497 patients [0.4%]). PDDI were reported for 2047 of 4831 patients (42.4%) at year 1, 1127 of 2751 patients (41.0%) at year 2, 761 of 1861 patients (40.9%) at year 3, 376 of 1058 patients (35.5%) at year 4, and 201 of 593 patients (33.9%) at year 5. Most of the PDDI were moderate with a proportion that increased from 60.4% (1237 of 2047 patients) to 80.6% (162 of 201 patients) between years 1 and 5. On the other hand, the occurrence of major PDDI decreased steadily over time, from 37.3% (763 of 2047 patients) at year 1 to 16.9% (34 of 201 patients) at year 5. Contraindicated combinations were identified in 0.1% to 1% (eTable 2 in the Supplement). The most prevalent drugs causing PDDI were selective serotonin reuptake inhibitors (paroxetine, fluoxetine, and duloxetine), antacids with sodium bicarbonate, and diosmectite. Drugs with the highest PDDI were ranked in Figure 3 and were classified according to the ATC drug class in eTable 3 in the Supplement.

In the AI cohort, 592 of 7437 patients (8.0%) had prescriptions with PDDI at baseline (Figure 2). Most of the PDDI were moderate (588 of 592 patients [99.3%]) and a small proportion were major (2 of 592 patients [0.3%]) or contraindicated (2 of 592 patients [0.3%]). PDDI reached 31.8% (2875 of 9031 patients) at year 1 and ranged between 31.4% (1802 of 5730 patients in year 2) and 32.8% (791 of 2411 patients in year 4) throughout the study period. PDDI were mainly moderate and varied between 94.7% (1230 of 1299 patients in year 3) and 99.3% (588 of 592 patients at baseline) whereas contraindicated combinations were identified in 0.2% (3 of 1802 patients in year 2) to 0.9% (4 of 435 patients in year 5) (eTable 2 in the Supplement). The most prevalent drugs causing PDDI were one of either antacids with sodium bicarbonate or diosmectite (Figure 3; eTable 3 in the Supplement).

### Adherence to Endocrine Therapy

According to MPR assessment, adherence to tamoxifen and AI was 79.3% (2824 of 3561 patients) and 88.8% (6479 of 7296 patients) at year 1 and reached 89.5% (426 of 476 patients) and 92.8% (1082 of 1166 patients) at year 5, respectively (Table 2). Among the patients that switched therapy, almost two-thirds of the switch occurred during year 1 (690 [63.6%] and 397 [60.5%] in the tamoxifen and AI, respectively). This proportion decreased steadily over the study period to 2.6% and 1.5% (28 and 10 patients, respectively) in the 2 cohorts. No association between adherence and PDDI was found, neither in the tamoxifen (OR, 0.99; 95% CI, 0.91-1.08) nor AI (OR, 1.05; 95% CI, 0.95-1.15) cohorts (Figure 3). In comparison to no PDDI, none of the PDDI categories showed a lower likelihood of adherence to ET (eTable 4 in the Supplement). In the tamoxifen cohort, higher odds of adherence were observed among adherent patients during the previous year (OR, 2.24; 95% CI, 1.95-2.56). In the AI cohort, lower odds of adherence were observed among patients with osteoarthritis (OR, 0.77; 95% CI, 0.66-0.90).

**Table 2.** Adherence by Cohort According to the Year of Treatment

Year	Patients, No. (%)	
	Tamoxifen adherence^a^	Aromatase inhibitor adherence^a^
1	2824 (79.3)	6479 (88.8)
2	1762 (84.7)	4332 (92.95)
3	1251 (87.7)	3018 (93.9)
4	762 (89.2)	1815 (93.5)
5	426 (89.5)	1082 (92.8)

^a^ Medication possession rate ≥80% defined the threshold for which patients were considered adherent to therapy.

### Comedications Relevant to the Management of Adverse Events

In the absence of granular data on the ET-associated adverse events, the comedications with drugs commonly prescribed to manage adverse events related to tamoxifen and AI were evaluated. In the tamoxifen cohort, the coprescription of nonsteroidal anti-inflammatory drugs increased by 5.9% after starting tamoxifen and remained stable over time. Similarly, the prescription of paracetamol-based combinations increased by 3.7% at year 1 and 6.5% at year 5. In the aromatase inhibitors cohort, the coprescription of nonsteroidal anti-inflammatory drugs varied between 40.6% at year 1 and 34.0% at year 5, compared with 36.8% during the last year before the prescription of ET. The prescription of paracetamol-based combinations increased by 3.7% at year 1 and 8.9% at year 5. The coprescription of duloxetine, venlafaxine and oxybutynin was inconsistent over time (eTable 5 and eFigure 2 in the Supplement).

## Discussion

Nonadherence to long-term medications is a multifaceted phenomenon in which multiple and complex determinants are involved, including patients’ attributes, disease characteristics, and treatment adverse effects.^26^ Because weak adherence to ET has been associated with lower breast cancer outcomes and increased direct and indirect health care costs, it is of utmost importance to identify the targetable determinants of nonadherence to set up suitable strategies to prevent ET discontinuation.^27,28^ Our study aimed to investigate whether PDDI between comedication and ET was associated with adherence to tamoxifen and AI.

By measuring adherence by MPR over 1-year prescription periods, we found that adherence remained relatively maintained over time among the patients who were prescribed ET, seemingly in contrast with prior published data which reported a decreased adherence to ET from years 1 to 5.^29,30^ However, this difference is only apparent owing to the timeframe used to assess treatment adherence. While prior studies measured adherence for the entire 5-year period and defined persistence by a treatment gap of less than 180 days between any 2 prescription dispenses, we used a more comprehensive approach that better reflects clinical practice by measuring MPR over 1-year prescription periods to better serve the purpose of this study, given that the proportion of PDDI changes over time (eTable 2 in the Supplement). Furthermore, the THIN database does not report the exact date of ET initiation and therefore the specific duration of ET cannot be computed accurately; to overcome this limitation, we limited the study population to patients with at least 1 year of continuous enrollment before and after the initiation of ET and applied a more stringent definition of persistence limited to less than 30 days gap between 2 prescription refills.

In line with published data, the present study showed that most of the PDDI with ET were moderate at baseline and increased from 6.9% to 27.3% between years 1 and 5, respectively.^18^ The majority of the associations presented moderate and major interactions and mainly included selective serotonin reuptake inhibitors in the tamoxifen cohort, and either antacids with sodium bicarbonate or diosmectite in the 2 cohorts. Paroxetine and fluoxetine are potent inhibitors of cytochrome P450 2D6 (CYP2D6) activity, which catalyzes the main pathway for converting tamoxifen into its most potent metabolite, endoxifen. A registry analysis of 2430 women with breast cancer has previously shown that the use of paroxetine during tamoxifen treatment results in 1 additional breast cancer death within 5 years of cessation of tamoxifen for every 19.7 patients so treated and that the risk with more extensive overlap was greater.^31^ More recently, 2 large population-based cohort studies showed that the concomitant use of tamoxifen and selective serotonin reuptake inhibitors did not increase the risk of death.^32,33^ On the other hand, the impact of antacids with sodium bicarbonate and intestinal adsorbent with diosmectite has not been fully evaluated.

Our findings showed that adherence was not associated with PDDI; conversely, higher odds of adherence were observed among previously adherent patients in the tamoxifen cohort and lower odds of adherence among patients with osteoarthritis in the AI cohort. In the first cohort, we found that adherent patients within the previous year had around 120% higher odds of adherence to tamoxifen. Indeed, tamoxifen-related adverse events are the major determinants for tamoxifen discontinuation, and thus adherent patients during the previous time period usually are those who experienced limited adverse events or learned to handle them and therefore are more likely to continue tamoxifen.^34,35,36,37,38^ In the AI cohort, patients with osteoarthritis had approximately 24% lower odds of adherence. Musculoskeletal symptoms are reported in more than 50% of patients receiving AI and joint symptoms may worsen during AI.^1,39^ We also found that users of oral diabetes medication had an approximately 37% higher odds of adherence to AI compared with nonusers (eFigure 3 in the Supplement). It is possible that this association is more related to patients’ specific behaviors in terms of medication uptake, so that those who are adherent to long-term medications, such as oral antidiabetics, are more likely to be adherent to other long-term treatments, including ET.^40^ In addition, metformin was shown to have a protective effect for musculoskeletal pain, which is the main adverse event reported with AI and thus potentially explains our findings.^41^ Finally, the prescription of medications commonly used to manage adverse events related to tamoxifen or AI did not significantly increase following the initiation of ET. We have previously shown that the majority of patients suffering from ET-related adverse events are unwilling to receive a second drug to treat these symptoms and are more prone to recur to complementary alternative medicines.^42,43^

### Limitations

Several limitations should also be acknowledged in the interpretation of this study. First, THIN cannot be considered a formal registry because it does not cover all the general practitioners in France; however, the selected physicians participating in the database are representative of the general practitioner population. Second, given the observation design of this study, we could not draw any causal conclusions. A third limitation is the missing data inherent to registry analysis. We opted for stringent inclusion criteria to ensure the exhaustiveness of data without any imputations but have led to a selection bias that limits the extrapolation of our findings. For instance, by including patients with at least 1 year of inclusion in the database and having consulted the general practitioner before and after the initiation of ET, we have excluded a proportion of patients that had PDDI and likely to be nonadherent to ET. Indeed, patients having regular follow-ups with their general practitioner are more likely to be adherent to their treatment and to present PDDI that are recognized at each annual visit whereas the PDDI of non-adherent patients are less likely to be identified. Moreover, the exclusion of patients that did not complete a 1-year follow up in the database may have led to the underrepresentation of the PDDI occurring within the first year. The stage of the disease during ET was not available; our efforts to find proxies to identify patients with adjuvant and metastatic indications, such as regular imaging evaluation reimbursement, were not successful. Survival outcomes were also lacking from the analysis. In addition, we were not able to retrieve over-the-counter medicines and complementary and alternative medicine, which are commonly used by patients as part of their medical care and share similar enzymatic pathways with ET thus increasing PDDI.^44^ Considering the study’s primary objective, the evaluation of adherence by MPR may be suboptimal but is the most commonly used and reproducible measure of adherence with administrative pharmacy dispensing data.^23^ For the purpose of this study, MPR was measured over 1-year intervals instead of the common methodology measuring adherence over the suggested ET duration. This methodology allowed us to evaluate the association of PDDI and concurring ET uptake, which will be probably underestimated if the MPR was measured over longer intervals. Alternatively, we did not choose a shorter time interval to evaluate adherence because we considered that most patients would visit their general practitioner once yearly.

## Conclusions

Adherence to ET in patients with breast cancer is a challenge given that factors into recurrence rates and survival. This study expanded on the understanding of nonadherence complexity, providing important insights on the prevalence of PDDI among patients receiving ET for breast cancer. Although PDDI were not significantly associated with adherence over time, these findings highlight the importance of a comprehensive medication assessment at each patient visit to avoid deleterious interactions that may negatively affect survival outcomes.

## Notes

Supplement.
eMethods.
**eTable 1.** Comedication Classes of Endocrine Therapy by Year **eTable 2.** Drug-drug Interactions With Endocrine Therapy **eTable 3.** Top 10 Drugs That Cause the Highest Number of Potential Drug-drug Interaction With Endocrine Therapy by Year **eTable 4.** Results of the Multivariable Model Analysis **eTable 5.** Comedication With Drugs Commonly Prescribed to Manage Adverse Events Related to Endocrine Therapy **eFigure 1.** Number of Patients With Coprescribed Medications During Years 1 to 5 **eFigure 2.** Proportion of Patients Receiving Treatments That Manage Adverse Events Potentially Caused by Endocrine Therapy **eFigure 3.** Sensitivity Analyses of the Multivariable Models of Adherence Adjusted for Age, Comedication, Adherence During the Previous Year, With and Without PDDI (Model 1 and 2, Respectively)

## References

[1] Condorelli R, Vaz-Luis I. Managing side effects in adjuvant endocrine therapy for breast cancer. Expert Rev Anticancer Ther. 2018;18(11):1101-1112. doi:10.1080/14737140.2018.1520096 30188738

[2] Robertson JFR, Paridaens RJ, Lichfield J, Bradbury I, Campbell C. Meta-analyses of phase 3 randomised controlled trials of third generation aromatase inhibitors versus tamoxifen as first-line endocrine therapy in postmenopausal women with hormone receptor-positive advanced breast cancer. Eur J Cancer. 2021;145:19-28. doi:10.1016/j.ejca.2020.11.038 33418233

[3] Brandão M, Maurer C, Ziegelmann PK, . Endocrine therapy-based treatments in hormone receptor-positive/HER2-negative advanced breast cancer: systematic review and network meta-analysis. ESMO Open. 2020;5(4):e000842. doi:10.1136/esmoopen-2020-000842 32847835 PMC7451473

[4] Smith KL, Stearns V. 54—Adjuvant Endocrine Therapy. In: Bland KI, Copeland EM, Klimberg VS, Gradishar WJ, eds. The Breast. 5th ed. Elsevier; 2018:736-751.e4, doi:10.1016/B978-0-323-35955-9.00054-4.

[5] Gennari A, André F, Barrios CH, ; ESMO Guidelines Committee. ESMO Clinical Practice Guideline for the diagnosis, staging and treatment of patients with metastatic breast cancer. Ann Oncol. 2021;32(12):1475-1495. doi:10.1016/j.annonc.2021.09.019 34678411

[6] Pistilli B, Paci A, Ferreira AR, . Serum detection of nonadherence to adjuvant tamoxifen and breast cancer recurrence risk. J Clin Oncol. 2020;38(24):2762-2772. doi:10.1200/JCO.19.01758 32568632 PMC7430219

[7] Hershman DL, Shao T, Kushi LH, . Early discontinuation and non-adherence to adjuvant hormonal therapy are associated with increased mortality in women with breast cancer. Breast Cancer Res Treat. 2011;126(2):529-537. doi:10.1007/s10549-010-1132-4 20803066 PMC3462663

[8] Kimmick G, Anderson R, Camacho F, Bhosle M, Hwang W, Balkrishnan R. Adjuvant hormonal therapy use among insured, low-income women with breast cancer. J Clin Oncol. 2009;27(21):3445-3451. doi:10.1200/JCO.2008.19.2419 19451445 PMC2717752

[9] Chlebowski RT, Kim J, Haque R. Adherence to endocrine therapy in breast cancer adjuvant and prevention settings. Cancer Prev Res (Phila). 2014;7(4):378-387. doi:10.1158/1940-6207.CAPR-13-0389 24441675 PMC11649036

[10] Neugut AI, Subar M, Wilde ET, . Association between prescription co-payment amount and compliance with adjuvant hormonal therapy in women with early-stage breast cancer. J Clin Oncol. 2011;29(18):2534-2542. doi:10.1200/JCO.2010.33.3179 21606426 PMC3138633

[11] Cluze C, Rey D, Huiart L, . Adjuvant endocrine therapy with tamoxifen in young women with breast cancer: determinants of interruptions vary over time. Ann Oncol. 2012;23(4):882-890. doi:10.1093/annonc/mdr330 21788360

[12] Murphy CC, Bartholomew LK, Carpentier MY, Bluethmann SM, Vernon SW. Adherence to adjuvant hormonal therapy among breast cancer survivors in clinical practice: a systematic review. Breast Cancer Res Treat. 2012;134(2):459-478. doi:10.1007/s10549-012-2114-5 22689091 PMC3607286

[13] Lash TL, Fox MP, Westrup JL, Fink AK, Silliman RA. Adherence to tamoxifen over the five-year course. Breast Cancer Res Treat. 2006;99(2):215-220. doi:10.1007/s10549-006-9193-0 16541307

[14] Garreau JR, Delamelena T, Walts D, Karamlou K, Johnson N. Side effects of aromatase inhibitors versus tamoxifen: the patients’ perspective. Am J Surg. 2006;192(4):496-498. doi:10.1016/j.amjsurg.2006.06.018 16978958

[15] Boonstra A, van Zadelhoff J, Timmer-Bonte A, Ottevanger PB, Beurskens CHG, van Laarhoven HWM. Arthralgia during aromatase inhibitor treatment in early breast cancer patients: prevalence, impact, and recognition by healthcare providers. Cancer Nurs. 2013;36(1):52-59. doi:10.1097/NCC.0b013e31824a7e18 22495502

[16] Mann E, Smith MJ, Hellier J, . Cognitive behavioural treatment for women who have menopausal symptoms after breast cancer treatment (MENOS 1): a randomised controlled trial. Lancet Oncol. 2012;13(3):309-318. doi:10.1016/S1470-2045(11)70364-3 22340966 PMC3314999

[17] Topaloğlu US, Özaslan E. Comorbidity and polypharmacy in patients with breast cancer. Breast Cancer. 2020;27(3):477-482. doi:10.1007/s12282-019-01040-8 31898155

[18] Domínguez-Alonso JA, Conde-Estévez D, Bosch D, Pi-Figueras M, Tusquets I. Breast cancer, placing drug interactions in the spotlight: is polypharmacy the cause of everything? Clin Transl Oncol. 2021;23(1):65-73. doi:10.1007/s12094-020-02386-8 32449126

[19] Mohamed MR, Ramsdale E, Loh KP, . Associations of polypharmacy and inappropriate medications with adverse outcomes in older adults with cancer: a systematic review and meta-analysis. Oncologist. 2020;25(1):e94-e108. doi:10.1634/theoncologist.2019-0406 31570516 PMC6964156

[20] Calip GS, Xing S, Jun DH, Lee WJ, Hoskins KF, Ko NY. Polypharmacy and adherence to adjuvant endocrine therapy for breast cancer. J Oncol Pract. 2017;13(5):e451-e462. doi:10.1200/JOP.2016.018317 28287854

[21] Blower P, de Wit R, Goodin S, Aapro M. Drug-drug interactions in oncology: why are they important and can they be minimized? Crit Rev Oncol Hematol. 2005;55(2):117-142. doi:10.1016/j.critrevonc.2005.03.007 15890526

[22] Riechelmann RP, Del Giglio A. Drug interactions in oncology: how common are they? Ann Oncol. 2009;20(12):1907-1912. doi:10.1093/annonc/mdp369 19713244

[23] Andrade SE, Kahler KH, Frech F, Chan KA. Methods for evaluation of medication adherence and persistence using automated databases. Pharmacoepidemiol Drug Saf. 2006;15(8):565-574. doi:10.1002/pds.1230 16514590

[24] Claude Bernard—La référence des produits de santé website. Accessed May 18, 2022. https://www.bcb.fr/

[25] Seabold S, Perktold J. Statsmodels: Econometric and statistical modeling with Python. *Proceedings of the 9th Python in Science Conference*. 2010;57:61.

[26] Osterberg L, Blaschke T. Adherence to medication. N Engl J Med. 2005;353(5):487-497. doi:10.1056/NEJMra050100 16079372

[27] Conn VS, Enriquez M, Ruppar TM, Chan KC. Meta-analyses of theory use in medication adherence intervention research. Am J Health Behav. 2016;40(2):155-171. doi:10.5993/AJHB.40.2.1 26931748 PMC4879970

[28] Hurtado-de-Mendoza A, Cabling ML, Lobo T, Dash C, Sheppard VB. Behavioral interventions to enhance adherence to hormone therapy in breast cancer survivors: a systematic literature review. Clin Breast Cancer. 2016;16(4):247-255.e3. doi:10.1016/j.clbc.2016.03.006 27133733 PMC4969158

[29] Partridge AH, Wang PS, Winer EP, Avorn J. Nonadherence to adjuvant tamoxifen therapy in women with primary breast cancer. J Clin Oncol. 2003;21(4):602-606. doi:10.1200/JCO.2003.07.071 12586795

[30] Cavazza M, Banks H, Ercolanoni M, . Factors influencing adherence to adjuvant endocrine therapy in breast cancer-treated women: using real-world data to inform a switch from acute to chronic disease management. Breast Cancer Res Treat. 2020;183(1):189-199. doi:10.1007/s10549-020-05748-6 32591986

[31] Kelly CM, Juurlink DN, Gomes T, . Selective serotonin reuptake inhibitors and breast cancer mortality in women receiving tamoxifen: a population based cohort study. BMJ. 2010;340:c693. doi:10.1136/bmj.c693 20142325 PMC2817754

[32] Donneyong MM, Bykov K, Bosco-Levy P, Dong YH, Levin R, Gagne JJ. Risk of mortality with concomitant use of tamoxifen and selective serotonin reuptake inhibitors: multi-database cohort study. BMJ. 2016;354:i5014. doi:10.1136/bmj.i5014 27694571 PMC5044871

[33] Valachis A, Garmo H, Weinman J, . Effect of selective serotonin reuptake inhibitors use on endocrine therapy adherence and breast cancer mortality: a population-based study. Breast Cancer Res Treat. 2016;159(2):293-303. doi:10.1007/s10549-016-3928-3 27492739 PMC5012147

[34] Demissie S, Silliman RA, Lash TL. Adjuvant tamoxifen: predictors of use, side effects, and discontinuation in older women. J Clin Oncol. 2001;19(2):322-328. doi:10.1200/JCO.2001.19.2.322 11208822

[35] Kahn KL, Schneider EC, Malin JL, Adams JL, Epstein AM. Patient centered experiences in breast cancer: predicting long-term adherence to tamoxifen use. Med Care. 2007;45(5):431-439. doi:10.1097/01.mlr.0000257193.10760.7f 17446829

[36] Owusu C, Buist DSM, Field TS, . Predictors of tamoxifen discontinuation among older women with estrogen receptor-positive breast cancer. J Clin Oncol. 2008;26(4):549-555. doi:10.1200/JCO.2006.10.1022 18071188

[37] Wouters H, Stiggelbout AM, Bouvy ML, . Endocrine therapy for breast cancer: assessing an array of women’s treatment experiences and perceptions, their perceived self-efficacy and nonadherence. Clin Breast Cancer. 2014;14(6):460-467.e2. doi:10.1016/j.clbc.2014.04.005 24981234

[38] Clancy C, Lynch J, OConnor P, Dowling M. Breast cancer patients’ experiences of adherence and persistence to oral endocrine therapy: a qualitative evidence synthesis. Eur J Oncol Nurs. 2020;44:101706. doi:10.1016/j.ejon.2019.101706 32007696

[39] Gaillard S, Stearns V. Aromatase inhibitor-associated bone and musculoskeletal effects: new evidence defining etiology and strategies for management. Breast Cancer Res. 2011;13(2):205. doi:10.1186/bcr2818 21457526 PMC3219175

[40] Yussof I, Mohd Tahir NA, Hatah E, Mohamed Shah N. Factors influencing five-year adherence to adjuvant endocrine therapy in breast cancer patients: a systematic review. Breast. 2022;62:22-35. doi:10.1016/j.breast.2022.01.012 35121501 PMC8818734

[41] Carvalho-E-Silva AP, Harmer AR, Ferreira ML, Ferreira PH. The effect of the anti-diabetic drug metformin on musculoskeletal pain: a cross-sectional study with 21,889 individuals from the UK biobank. Eur J Pain. 2021;25(6):1264-1273. doi:10.1002/ejp.1747 33561890

[42] Yuanqing P, Yong T, Haiqian L, . Acupuncture for hormone therapy-related side effects in breast cancer patients: a GRADE-assessed systematic review and updated meta-analysis. Integr Cancer Ther. 2020;19:1534735420940394. doi:10.1177/1534735420940394 32718258 PMC7388099

[43] Akla S, Rassy E, Di-Meglio A, . Patient’s point of view on how to promote adherence to adjuvant endocrine therapy (ET): a large French survey. Cancer Res. 2022;82(4_Supplement):P4-11-24. doi:10.1158/1538-7445.SABCS21-P4-11-24

[44] Trivedi R, Salvo MC. Utilization and safety of common over-the-counter dietary/nutritional supplements, herbal agents, and homeopathic compounds for disease prevention. Med Clin North Am. 2016;100(5):1089-1099. doi:10.1016/j.mcna.2016.04.017 27542428

