# Supplementary material for: Association of Adherence to Endocrine Therapy Among Patients With Breast Cancer and Potential Drug-Drug Interactions
Source: JAMA Netw Open. 2022 Dec 2;5(12):e2244849. doi: 10.1001/jamanetworkopen.2022.44849 (PMC9719053; doi:10.1001/jamanetworkopen.2022.44849)
Supplement: Supplement. — eMethods. eTable 1. Comedication Classes of Endocrine Therapy by Year eTable 2. Drug-drug Interactions With Endocrine Therapy eTable 3. Top 10 Drugs That Cause the Highest Number of Potential Drug-drug Interaction With Endocrine Therapy by Year eTable 4. Results of the Multivariable Model Analysis eTable 5. Comedication With Drugs Commonly Prescribed to Manage Adverse Events Related to Endocrine Therapy eFigure 1. Number of Patients With Coprescribed Medications During Years 1 to 5 eFigure 2. Proportion of Patients Receiving Treatments That Manage Adverse Events Potentially Caused by Endocrine Therapy eFigure 3. Sensitivity Analyses of the Multivariable Models of Adherence Adjusted for Age, Comedication, Adherence During the Previous Year, With and Without PDDI (Model 1 and 2, Respectively) [file jamanetwopen-e2244849-s001.pdf]

Supplemental Online Content

Rassy E, Bardet A, Bougacha O, et al. Association of adherence to endocrine therapy among patients with breast cancer and potential drug-drug interactions. *JAMA Netw Open.* 2022;5(12):e2244849. doi:10.1001/jamanetworkopen.2022.44849

eMethods.

eTable 1. Comedication Classes of Endocrine Therapy by Year

eTable 2. Drug-drug Interactions With Endocrine Therapy

eTable 3. Top 10 Drugs That Cause the Highest Number of Potential Drug-drug Interaction With Endocrine Therapy by Year

eTable 4. Results of the Multivariable Model Analysis

eTable 5. Comedication With Drugs Commonly Prescribed to Manage Adverse Events Related to Endocrine Therapy

eFigure 1. Number of Patients With Coprescribed Medications During Years 1 to 5

eFigure 2. Proportion of Patients Receiving Treatments That Manage Adverse Events Potentially Caused by Endocrine Therapy

eFigure 3. Sensitivity Analyses of the Multivariable Models of Adherence Adjusted for Age, Comedication, Adherence During the Previous Year, With and Without PDDI (Model 1 and 2, Respectively)

© 2022 Rassy E et al. *JAMA Network Open.*

eMethods

Study design

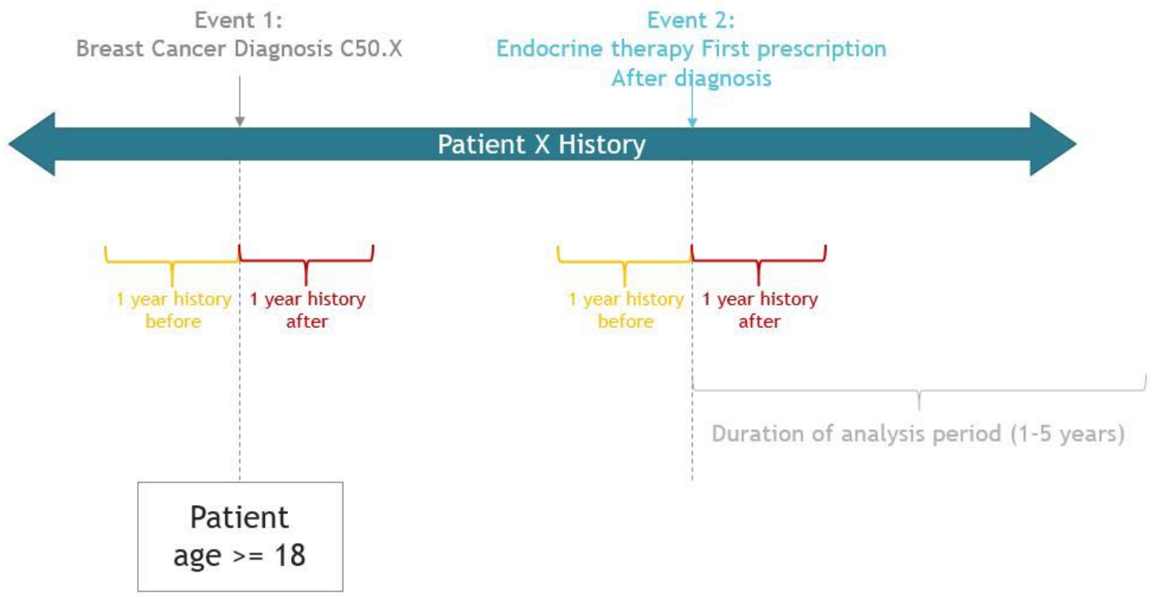

Data collection

- Patients from Reimbursement Data

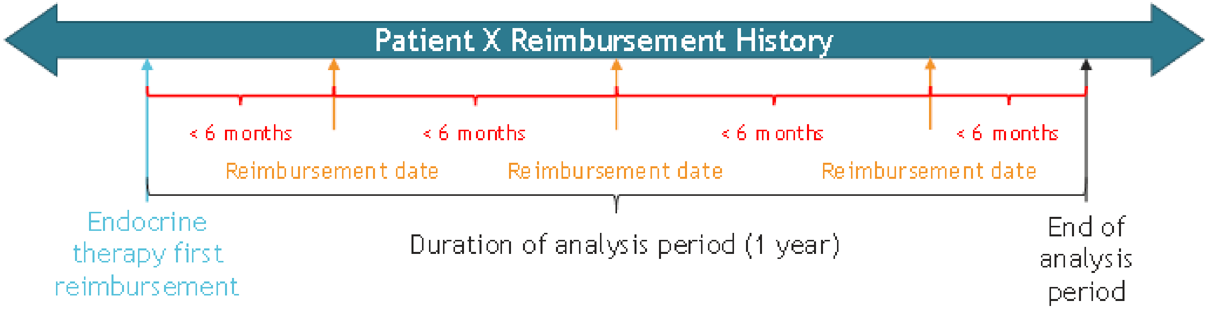

- Patients from Prescription Data

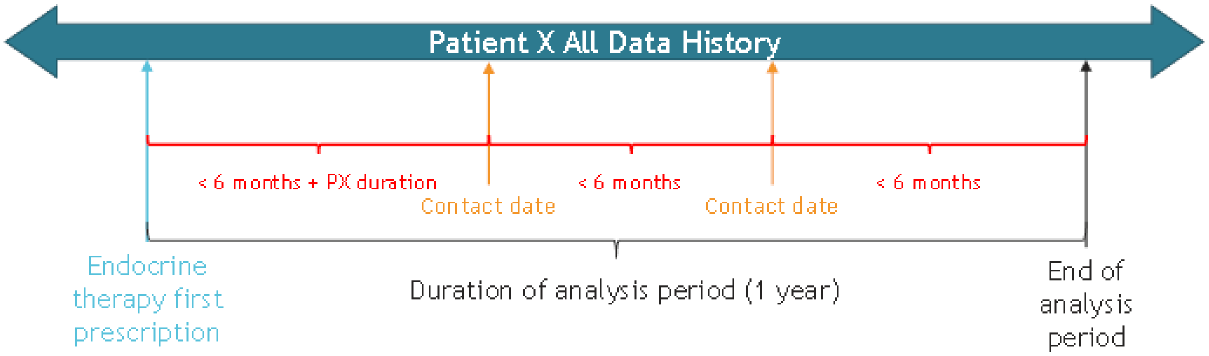

© 2022 Rassy E et al. *JAMA Network Open.*

Computation of medication possession ratio

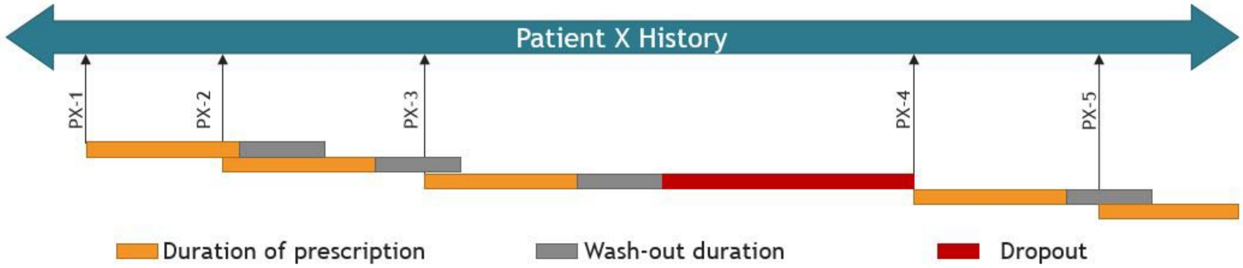

PX	PX-Date	PX-Duration	Return-Date	Real-Duration	Max-Admissible-Date	Valid?	MPR
1	2017-01-01	30	2017-01-28	27	2017-03-02	1	107%
2	2017-01-28	30	2017-03-14	45	2017-03-29	1	66,66%
3	2017-03-14	30	2017-06-22	100	2017-05-13	0	-
4	2017-06-22	30	2017-08-01	40	2017-08-21	1	75%
5	2017-08-01	30	-	-	-	-	-

$$MPR_{Final} = \frac{\sum PX-Duration}{\sum Real-Duration} = \frac{30+30+30}{27+45+40} = \frac{90}{112} = 80{,}35\%$$

© 2022 Rassy E et al. *JAMA Network Open.*

eTable 1. Comedication Classes of Endocrine Therapy by Year

eTable 1A: Comedication classes at baseline* of endocrine therapy

Drug Category	Frequency of taking	Tamoxifen cohort (3,564 patients) Patients, No. (%)	Aromatase inhibitor cohort (7,299 patients) Patients, No. (%)
Antidepressants	Nonuse	2,787 (78.2)	5,812 (79.6)
	Infrequent use	330 (9.3)	504 (6.9)
	Frequent use	447 (12.5)	983 (13.5)
Antihypertensive	Nonuse	3,504 (98.3)	7,075 (96.9)
	Infrequent use	22 (0.6)	54 (0.7)
	Frequent use	38 (1.1)	170 (2.3)
Antipsychotics	Nonuse	3,411 (95.7)	7,065 (96.8)
	Infrequent use	90 (2.5)	84 (1.2)
	Frequent use	63 (1.8)	150 (2.1)
Anxiolytics	Nonuse	2,309 (64.8)	4,752 (65.1)
	Infrequent use	694 (19.5)	1,247 (17.1)
	Frequent use	561 (15.7)	1,300 (17.8)
Insulin analogs	Nonuse	3,530 (99.0)	7,155 (98.0)
	Infrequent use	11 (0.3)	25 (0.3)
	Frequent use	23 (0.6)	119 (1.6)
Oral diabetes medications	Nonuse	3,406 (95.6)	6,505 (89.1)
	Infrequent use	32 (0.9)	134 (1.8)
	Frequent use	126 (3.5)	660 (9.0)
Lipid Modifying	Nonuse	3,136 (88.0)	5,007 (68.6)
	Infrequent use	103 (2.9)	459 (6.3)
	Frequent use	325 (9.1)	1,833 (25.1)
Opioid containing analgesics	Nonuse	2,309 (64.8)	5,401 (74.0)
	Infrequent use	694 (19.5)	1,296 (17.8)
	Frequent use	561 (15.7)	602 (8.2)

* Within1 year before the first prescription of endocrine therapy.

© 2022 Rassy E et al. *JAMA Network Open.*

eTable 1B: Comedication classes at year 1 of endocrine therapy			
Drug Category	Frequency of taking	Tamoxifen cohort (3,564 patients) Patients, No. (%)	Aromatase inhibitor cohort (7,299 patients) Patients, No. (%)
Antidepressants	Nonuse	2,679 (75.2)	5,612 (76.9)
	Infrequent use	264 (7.4)	383 (5.2)
	Frequent use	621 (17.4)	1,304 (17.9)
Antihypertensive	Nonuse	3,503 (98.3)	7,052 (96.6)
	Infrequent use	15 (0.4)	38 (0.5)
	Frequent use	46 (1.3)	209 (2.9)
Antipsychotics	Nonuse	3,420 (96.0)	7,041 (96.5)
	Infrequent use	61 (1.7)	66 (0.9)
	Frequent use	83 (2.3)	192 (2.6)
Anxiolytics	Nonuse	2,411 (67.6)	4,915 (67.3)
	Infrequent use	515 (14.5)	814 (11.2)
	Frequent use	638 (17.9)	1,570 (21.5)
Insulin analogs	Nonuse	3,525 (98.9)	7,123 (97.6)
	Infrequent use	5 (0.1)	17 (0.2)
	Frequent use	34 (1.0)	159 (2.2)
Oral diabetes medications	Nonuse	3,367 (94.5)	6,421 (88.0)
	Infrequent use	20 (0.6)	66 (0.9)
	Frequent use	177 (5.0)	812 (11.1)
Lipid Modifying	Nonuse	3,091 (86.7)	4,848 (66.4)
	Infrequent use	82 (2.3)	271 (3.7)
	Frequent use	391 (11.0)	2,180 (29.9)
Opioid containing analgesics	Nonuse	2,716 (76.2)	5,586 (76.5)
	Infrequent use	555 (15.6)	941 (12.9)
	Frequent use	293 (8.2)	772 (10.6)

© 2022 Rassy E et al. *JAMA Network Open.*

eTable 1C: Comedication classes at year 2 of endocrine therapy			
Drug Category	Frequency of taking	Tamoxifen cohort (2,081 patients) Patients, No. (%)	Aromatase inhibitor cohort (4,661 patients) Patients, No. (%)
Antidepressants	Nonuse	1,582 (76.0)	3,575 (76.7)
	Infrequent use	127 (6.1)	215 (4.6)
	Frequent use	372 (17.9)	871 (18.7)
Antihypertensive	Nonuse	2,043 (98.2)	4,472 (95.9)
	Infrequent use	6 (0.3)	32 (0.7)
	Frequent use	32 (96.4)	157 (3.4)
Antipsychotics	Nonuse	2,006 (96.4)	4,505 (96.7)
	Infrequent use	21 (1.0)	27 (0.6)
	Frequent use	54 (2.6)	129 (2.8)
Anxiolytics	Nonuse	1,478 (71.0)	3,144 (67.5)
	Infrequent use	242 (11.6)	514 (11.0)
	Frequent use	361 (17.3)	1,003 (21.5)
Insulin analogs	Nonuse	2,056 (98.8)	4,541 (97.4)
	Infrequent use	5 (0.2)	9 (0.2)
	Frequent use	20 (1.0)	111 (2.4)
Oral diabetes medications	Nonuse	1,925 (92.5)	4,008 (86.0)
	Infrequent use	15 (0.7)	41 (0.9)
	Frequent use	141 (6.8)	612 (13.1)
Lipid Modifying	Nonuse	1,766 (84.9)	2,931 (62.9)
	Infrequent use	43 (2.1)	195 (4.2)
	Frequent use	272 (13.1)	1,535 (32.9)
Opioid containing analgesics	Nonuse	1,603 (77.0)	3,640 (78.1)
	Infrequent use	292 (14.0)	523 (11.2)
	Frequent use	186 (8.9)	498 (10.7)

© 2022 Rassy E et al. *JAMA Network Open.*

eTable 1D: Comedication classes at year 3 of endocrine therapy			
Drug Category	Frequency of taking	Tamoxifen cohort (1,426 patients) Patients, No. (%)	Aromatase inhibitor cohort (3,214 patients) Patients, No. (%)
Antidepressants	Nonuse	1,084 (76.0)	2,471 (76.9)
	Infrequent use	100 (7.0)	137 (4.3)
	Frequent use	242 (17.0)	606 (18.9)
Antihypertensive	Nonuse	1,399 (98.1)	3,083 (95.9)
	Infrequent use	8 (0.6)	20 (0.6)
	Frequent use	19 (1.3)	111 (3.5)
Antipsychotics	Nonuse	1,370 (96.1)	3,090 (96.1)
	Infrequent use	19 (1.3)	28 (0.9)
	Frequent use	37 (2.6)	96 (3.0)
Anxiolytics	Nonuse	972 (68.2)	2,191 (68.2)
	Infrequent use	176 (12.3)	328 (10.2)
	Frequent use	278 (19.5)	695 (21.6)
Insulin analogs	Nonuse	1,409 (98.8)	3,121 (97.1)
	Infrequent use	3 (0.2)	13 (0.4)
	Frequent use	14 (1.0)	80 (2.5)
Oral diabetes medications	Nonuse	1,304 (91.4)	2,714 (84.4)
	Infrequent use	7 (0.5)	44 (1.4)
	Frequent use	115 (8.1)	456 (14.2)
Lipid Modifying	Nonuse	1,177 (82.5)	1,962 (61.0)
	Infrequent use	51 (3.6)	156 (4.9)
	Frequent use	198 (13.9)	1,096 (34.1)
Opioid containing analgesics	Nonuse	1,078 (75.6)	2,527 (78.6)
	Infrequent use	195 (13.7)	345 (10.7)
	Frequent use	153 (10.7)	342 (10.6)

© 2022 Rassy E et al. *JAMA Network Open.*

eTable 1E: Comedication classes at year 4 of endocrine therapy			
Drug Category	Frequency of taking	Tamoxifen cohort (854 patients) Patients, No. (%)	Aromatase inhibitor cohort (1,943 patients) Patients, No. (%)
Antidepressants	Nonuse	659 (77.2)	1,498 (77.1)
	Infrequent use	43 (5.0)	67 (3.4)
	Frequent use	152 (17.8)	378 (19.5)
Antihypertensive	Nonuse	834 (97.7)	1,864 (95.9)
	Infrequent use	7 (0.8)	6 (0.3)
	Frequent use	13 (1.5)	73 (3.8)
Antipsychotics	Nonuse	831 (97.3)	1,882 (96.9)
	Infrequent use	5 (0.6)	10 (0.5)
	Frequent use	18 (2.1)	51 (2.6)
Anxiolytics	Nonuse	564 (66.0)	1,325 (68.2)
	Infrequent use	109 (12.8)	180 (9.3)
	Frequent use	181 (21.2)	438 (22.5)
Insulin analogs	Nonuse	841 (98.5)	1,892 (97.4)
	Infrequent use	1 (0.1)	6 (0.3)
	Frequent use	12 (1.4)	45 (2.3)
Oral diabetes medications	Nonuse	768 (89.9)	1,625 (83.6)
	Infrequent use	7 (0.8)	18 (0.9)
	Frequent use	79 (9.3)	300 (15.4)
Lipid Modifying	Nonuse	687 (80.4)	1,131 (58.2)
	Infrequent use	14 (1.6)	88 (4.5)
	Frequent use	153 (17.9)	724 (37.3)
Opioid containing analgesics	Nonuse	637 (74.6)	1,525 (78.5)
	Infrequent use	130 (15.2)	192 (9.9)
	Frequent use	87 (10.2)	226 (11.6)

© 2022 Rassy E et al. *JAMA Network Open.*

eTable 1F: Comedication classes at year 5 of endocrine therapy			
Drug Category	Frequency of taking	Tamoxifen cohort (476 patients) Patients, No. (%)	Aromatase inhibitor cohort (1,082 patients) Patients, No. (%)
Antidepressants	Nonuse	376 (79.0)	827 (76.4)
	Infrequent use	21 (4.4)	34 (3.1)
	Frequent use	79 (16.6)	221 (20.4)
Antihypertensive	Nonuse	465 (97.7)	1,018 (94.1)
	Infrequent use	2 (0.4)	12 (1.1)
	Frequent use	9 (1.9)	52 (4.8)
Antipsychotics	Nonuse	465 (97.7)	1,050 (97.0)
	Infrequent use	2 (0.4)	7 (0.6)
	Frequent use	9 (1.9)	25 (2.3)
Anxiolytics	Nonuse	323 (67.9)	727 (67.2)
	Infrequent use	45 (9.5)	108 (10.0)
	Frequent use	108 (22.7)	247 (22.8)
Insulin analogs	Nonuse	469 (98.5)	1,059 (97.9)
	Infrequent use	0 (0)	1 (0.1)
	Frequent use	7 (1.5)	22 (2.0)
Oral diabetes medications	Nonuse	425 (89.3)	899 (83.1)
	Infrequent use	6 (1.3)	4 (0.4)
	Frequent use	45 (9.5)	179 (16.5)
Lipid Modifying	Nonuse	371 (77.9)	625 (57.8)
	Infrequent use	7 (1.5)	25 (2.3)
	Frequent use	98 (20.6)	432 (39.9)
Opioid containing analgesics	Nonuse	349 (73.3)	848 (78.4)
	Infrequent use	73 (15.3)	107 (9.9)
	Frequent use	54 (11.3)	127 (11.7)

© 2022 Rassy E et al. *JAMA Network Open.*

eTable 2. Drug-drug Interactions With Endocrine Therapy

eTable 2A: Potential drug-drug interactions with endocrine therapy at baseline and during follow up			
Potential drug-drug interaction		Tamoxifen cohort Patients, No. (%)	Aromatase inhibitor cohort Patients, No. (%)
Potential drug-drug interaction at baseline	Not detected	3,173 (86.5)	6,845 (92.0)
	Minor	14 (0.4)	0 (0)
	Moderate	254 (6.9)	588 (7.9)
	Major	227 (6.2)	2 (0.1)
	Contraindicated	2 (0.1)	2 (0.1)
Potential drug-drug interaction at follow up year 1	Not detected	2,784 (57.6)	6,156 (68.2)
	Minor	41 (0.8)	6 (0.1)
	Moderate	1,237 (25.6)	2,811 (31.1)
	Major	763 (15.8)	50 (0.6)
	Contraindicated	6 (0.1)	8 (0.1)
Potential drug-drug interaction at follow up year 2	Not detected	1,624 (59.0)	3,928 (68.6)
	Minor	28 (1.0)	2 (0.1)
	Moderate	758 (27.6)	1,730 (30.2)
	Major	340 (12.4)	67 (1.2)
	Contraindicated	1 (0.1)	3 (0.1)
Potential drug-drug interaction at follow up year 3	Not detected	1,100 (59.1)	2,663 (67.2)
	Minor	31 (1.7)	1 (0.1)
	Moderate	521 (28.0)	1,230 (31.0)
	Major	206 (11.1)	65 (1.6)
	Contraindicated	3 (0.2)	3 (0.1)
Potential drug-drug interaction at follow up year 4	Not detected	682 (64.5)	1,620 (67.2)
	Minor	10 (0.9)	0 (0)
	Moderate	281 (26.6)	780 (32.4)
	Major	85 (8.0)	9 (0.4)
	Contraindicated	0 (0)	2 (0.1)
Potential drug-drug interaction at follow up year 5	Not detected	392 (66.1)	905 (67.5)
	Minor	3 (0.5)	0 (0)
	Moderate	162 (27.3)	425 (31.7)
	Major	34 (5.7)	6 (0.4)
	Contraindicated	2 (0.3)	4 (0.3)
* Within1 year before the first prescription of endocrine therapy.			

© 2022 Rassy E et al. *JAMA Network Open.*

eTable 2B: Patients with potential drug-drug interactions with endocrine therapy at baseline and during follow up			
Potential drug-drug interaction		Tamoxifen cohort Patients, No. (%)	Aromatase inhibitor cohort Patients, No. (%)
Potential drug-drug interaction at baseline	Not detected	3,173 (89.0)	6,845 (93.8)
	Minor	10 (0.3)	0 (0)
	Moderate	199 (5.6)	450 (6.2)
	Major	180 (5.1)	2 (0.1)
	Contraindicated	2 (0.1)	2 (0.1)
Potential drug-drug interaction at follow up year 1	Not detected	2,796 (78.5)	6,181 (84.7)
	Minor	14 (0.4)	1 (0.1)
	Moderate	459 (12.9)	1,093 (15.0)
	Major	291 (8.2)	22 (0.3)
	Contraindicated	4 (0.1)	2 (0.1)
Potential drug-drug interaction at follow up year 2	Not detected	1,624 (78.0)	3,928 (84.3)
	Minor	8 (0.4)	1 (0.1)
	Moderate	302 (14.5)	707 (15.2)
	Major	146 (7.0)	23 (0.5)
	Contraindicated	1 (0.1)	2 (0.1)
Potential drug-drug interaction at follow up year 3	Not detected	1,100 (77.1)	2,663 (82.9)
	Minor	8 (0.6)	1 (0.1)
	Moderate	236 (16.5)	535 (16.6)
	Major	81 (5.7)	14 (0.4)
	Contraindicated	1 (0.1)	1 (0.1)
Potential drug-drug interaction at follow up year 4	Not detected	682 (79.9)	1,620 (83.4)
	Minor	2 (0.2)	0 (0)
	Moderate	132 (15.5)	316 (16.3)
	Major	38 (4.4)	5 (0.3)
	Contraindicated	0 (0)	2 (0.1)
Potential drug-drug interaction at follow up year 5	Not detected	392 (82.4)	905 (83.6)
	Minor	1 (0.2)	0 (0)
	Moderate	70 (14.7)	170 (15.7)
	Major	12 (2.5)	6 (0.6)
	Contraindicated	1 (0.2)	1 (0.1)

* Within1 year before the first prescription of endocrine therapy.

© 2022 Rassy E et al. *JAMA Network Open.*

eTable 3. Top 10 Drugs That Cause the Highest Number of Potential Drug-drug Interaction With Endocrine Therapy by Year

eTable 3A: Top 10 drugs that causes the highest number of potential drug-drug interactions with endocrine therapy at baseline			
Endocrine therapy	Drug	ATC drug class	Number of interactions
Tamoxifen	Paroxetine	N06AB05	98
	Antacids with sodium bicarbonate	A02AH	97
	Fluoxetine	N06AB03	83
	Diosmectite	A07BC05	44
	Duloxetine	N06AX21	32
	Fluindione	B01AA12	31
	Ordinary salt combinations and antiflatulents	A02AF02	12
	Medicinal charcoal, combinations	A07BA51	12
	Antacids, other combinations	A02AX	10
	Magnesium pidolate	A12CC08	10
Aromatase inhibitors	Antacids with sodium bicarbonate	A02AH	308
	Diosmectite	A07BC05	126
	Antacids, other combinations	A02AX	34
	Ordinary salt combinations and antiflatulents	A02AF02	18
	Ordinary salt combinations	A02AD01	17
	Sodium picosulfate, combinations	A06AB58	17
	Polystyrene sulfonate	V03AE01	12
	Medicinal charcoal, combinations	A07BA51	11
	Colestyramine	C10AC01	11
	Magnesium pidolate	A12CC08	10

* Within1 year before first prescription of endocrine therapy.

ATC: Anatomical Therapeutic Chemical

© 2022 Rassy E et al. *JAMA Network Open.*

eTable 3B: Top 10 drugs that causes the highest number of potential drug-drug interactions with endocrine therapy at follow up year 1			
Endocrine therapy	Drug	ATC drug class	Number of interactions
Tamoxifen	Antacids with sodium bicarbonate	A02AH	415
	Diosmectite	A07BC05	390
	Paroxetine	N06AB05	284
	Fluoxetine	N06AB03	254
	Duloxetine	N06AX21	163
	Ordinary salt combinations and antiflatulents	A02AF02	83
	Terbinafine	D01BA02	82
	Fluindione	B01AA12	66
	Antacids, other combinations	A02AX	53
	Ordinary salt combinations	A02AD01	49
Aromatase inhibitors	Antacids with sodium bicarbonate	A02AH	1224
	Diosmectite	A07BC05	816
	Ordinary salt combinations and antiflatulents	A02AF02	159
	Antacids, other combinations	A02AX	135
	Sodium picosulfate, combinations	A06AB58	130
	Ordinary salt combinations	A02AD01	91
	Polystyrene sulfonate	V03AE01	50
	Medicinal charcoal, combinations	A07BA51	43
	Aluminium phosphate	A02AB03	37
	Magnesium pidolate	A12CC08	35
ATC: Anatomical Therapeutic Chemical			

© 2022 Rassy E et al. *JAMA Network Open.*

eTable 3C: Top 10 drugs that causes the highest number of potential drug-drug interactions with endocrine therapy at follow up year 2			
Endocrine therapy	Drug	ATC drug class	Number of interactions
Tamoxifen	Antacids with sodium bicarbonate	A02AH	275
	Diosmectite	A07BC05	198
	Paroxetine	N06AB05	172
	Fluoxetine	N06AB03	113
	Duloxetine	N06AX21	80
	Antacids, other combinations	A02AX	41
	Ordinary salt combinations and antiflatulents	A02AF02	36
	Fluindione	B01AA12	34
	Medicinal charcoal, combinations	A07BA51	31
	Terbinafine	D01BA02	26
Aromatase inhibitors	Antacids with sodium bicarbonate	A02AH	843
	Diosmectite	A07BC05	464
	Ordinary salt combinations and antiflatulents	A02AF02	99
	Antacids, other combinations	A02AX	79
	Ordinary salt combinations	A02AD01	59
	Sodium picosulfate, combinations	A06AB58	49
	Polystyrene sulfonate	V03AE01	36
	Medicinal charcoal, combinations	A07BA51	28
	Magnesium pidolate	A12CC08	19
	Aluminium phosphate	A02AB03	18
ATC: Anatomical Therapeutic Chemical			

© 2022 Rassy E et al. *JAMA Network Open.*

eTable 3D: Top 10 drugs that causes the highest number of potential drug-drug interactions with endocrine therapy at follow up year 3			
Endocrine therapy	Drug	ATC drug class	Number of interactions
Tamoxifen	Diosmectite	A07BC05	166
	Antacids with sodium bicarbonate	A02AH	130
	Fluoxetine	N06AB03	128
	Paroxetine	N06AB05	63
	Ordinary salt combinations and antiflatulents	A02AF02	51
	Duloxetine	N06AX21	46
	Terbinafine	D01BA02	28
	Antacids, other combinations	A02AX	21
	Fluindione	B01AA12	20
Aromatase inhibitors	Antacids with sodium bicarbonate	A02AH	592
	Diosmectite	A07BC05	418
	Ordinary salt combinations and antiflatulents	A02AF02	78
	Antacids, other combinations	A02AX	50
	Sodium picosulfate, combinations	A06AB58	38
	Ordinary salt combinations	A02AD01	32
	Medicinal charcoal, combinations	A07BA51	21
	Polystyrene sulfonate	V03AE01	14
	Colestyramine	C10AC01	13
	Aluminium phosphate	A02AB03	13
ATC: Anatomical Therapeutic Chemical			

© 2022 Rassy E et al. *JAMA Network Open.*

eTable 3E: Top 10 drugs that causes the highest number of potential drug-drug interactions with endocrine therapy at follow up year 4			
Endocrine therapy	Drug	ATC drug class	Number of interactions
Tamoxifen	Antacids with sodium bicarbonate	A02AH	77
	Diosmectite	A07BC05	68
	Paroxetine	N06AB05	30
	Fluoxetine	N06AB03	28
	Duloxetine	N06AX21	24
	Sodium picosulfate, combinations	A06AB58	17
	Fluindione	B01AA12	14
	Ordinary salt combinations and antiflatulents	A02AF02	12
	Terbinafine	D01BA02	11
	Antacids, other combinations	A02AX	7
Aromatase inhibitors	Antacids with sodium bicarbonate	A02AH	363
	Diosmectite	A07BC05	251
	Ordinary salt combinations and antiflatulents	A02AF02	56
	Antacids, other combinations	A02AX	34
	Sodium picosulfate, combinations	A06AB58	29
	Ordinary salt combinations	A02AD01	26
	Medicinal charcoal, combinations	A07BA51	18
	Magnesium pidolate	A12CC08	14
	Aluminium phosphate	A02AB03	12
	Polystyrene sulfonate	V03AE01	9
ATC: Anatomical Therapeutic Chemical			

eTable 3F: Top 10 drugs that causes the highest number of potential drug-drug interactions with endocrine therapy at follow up year 5			
Endocrine therapy	Drug	ATC drug class	Number of interactions
Tamoxifen	Antacids with sodium bicarbonate	A02AH	40
	Diosmectite	A07BC05	35
	Paroxetine	N06AB05	22
	Duloxetine	N06AX21	11
	Fluindione	B01AA12	10
	Ordinary salt combinations and antiflatulents	A02AF02	9
	Fluoxetine	N06AB03	7
	Sodium picosulfate, combinations	A06AB58	6
	Polystyrene sulfonate	V03AE02	6
	Antacids, other combinations	A02AX	4
Aromatase inhibitors	Antacids with sodium bicarbonate	A02AH	192
	Diosmectite	A07BC05	132
	Ordinary salt combinations and antiflatulents	A02AF02	51
	Antacids, other combinations	A02AX	20
	Sodium picosulfate, combinations	A06AB58	18
	Ordinary salt combinations	A02AD01	14
	Magnesium pidolate	A12CC08	8
	Medicinal charcoal, combinations	A07BA51	5
	Aluminium hydroxide	A02AB01	4
	Other drugs for peptic ulcer and gastro-oesophageal reflux disease	A02BX	4
ATC: Anatomical Therapeutic Chemical			

eTable 4. Results of the Multivariable Model Analysis

eTable 4 : Results of the multivariable model analysis			
		Tamoxifen cohort<br>OR (95% CI)	Aromatase inhibitor cohort<br>OR (95% CI)
Age		1.022 (1.02-1.025)	1.03 (1.028-1.032)
Coronary artery disease		0.383 (0.104-1.414)	0.777 (0.348-1.734)
Hypertension		1.114 (0.934-1.329)	1.092 (0.943-1.264)
Diabetes mellitus		0.82 (0.586-1.149)	1.119 (0.903-1.387)
Thyroid dysfunction		8.212 (1.063-63.448)	2.709 (0.692-10.605)
Rheumatologic disease		0.759 (0.416-1.385)	**1.667 (1.029-2.702)**
Osteoarthritis		1.049 (0.829-1.328)	**0.77 (0.656-0.903)**
Epilepsy		0.9 (0.39-2.08)	1.259 (0.678-2.34)
Dementia		0.286 (0.037-2.201)	0.548 (0.146-2.052)
Cerebrovascular disease		0.83 (0.535-1.286)	0.959 (0.698-1.318)
COPD		0.751 (0.423-1.335)	1.265 (0.782-2.047)
Asthma		1.06 (0.845-1.33)	0.986 (0.799-1.218)
Depression		0.984 (0.839-1.154)	0.939 (0.813-1.085)
Other cancers		1.017 (0.809-1.279)	0.98 (0.805-1.193)
Previous MPR ≥ 80%		**2.235 (1.951-2.56)**	1.047 (0.962-1.138)
PDDI		0.991 (0.914-1.075)	1.046 (0.954-1.147)
	Absent	Reference	Reference
	Minor	0.74 (0.02-3.12)	-
	Moderate	1.15 (0.73-1.38)	1.01 (0.77-1.28)
	Major	1.06 (0.78-1.33)	0.59 (0.01-2.4)
	Contraindicated	0.79 (0.09-3.26)	0.18 (0.001-1.01)

CI: confidence interval; COPD : chronic obstructive pulmonary disease; OR: odds ratio; PDDI : potential-drug-drug interactions

© 2022 Rassy E et al. *JAMA Network Open.*

eTable 5. Comedication With Drugs Commonly Prescribed to Manage Adverse Events Related to Endocrine Therapy

eTable 5: Comedication with drugs commonly prescribed to manage adverse events related to endocrine therapy			
		Tamoxifen cohort (%)	Aromatase inhibitor cohort (%)
Nonsteroidal anti-inflammatory drugs	Baseline*	38.3	36.8
	First Year	44.2	40.6
	Second Year	42.3	38.1
	Third Year	44.1	36.7
	Fourth Year	44.3	35.6
	Fifth Year	44.5	34.0
Paracetamol based combinations	Baseline*	61.8	67.4
	First Year	65.5	71.1
	Second Year	67.5	73.9
	Third Year	69.7	73.0
	Fourth Year	67.8	74.6
	Fifth Year	68.3	76.3
Duloxetine	Baseline*	0.89	0.8
	First Year	1.12	1.12
	Second Year	1.34	1.26
	Third Year	1.42	1.47
	Fourth Year	1.52	1.54
	Fifth Year	1.26	1.66
Venlafaxine	Baseline*	3.25	2.75
	First Year	4.0	3.5
	Second Year	4.32	3.49
	Third Year	4.63	3.38
	Fourth Year	4.1	2.98
	Fifth Year	2.94	4.15
Oxybutynin	Baseline*	0.95	0.71
	First Year	1.12	1.12
	Second Year	1.39	0.79
	Third Year	1.28	0.97
	Fourth Year	1.52	0.92
	Fifth Year	1.05	0.64
* Within1 year before the first prescription of endocrine therapy.			

© 2022 Rassy E et al. *JAMA Network Open.*

eFigure 1. Number of Patients With Coprescribed Medications During Years 1 to 5

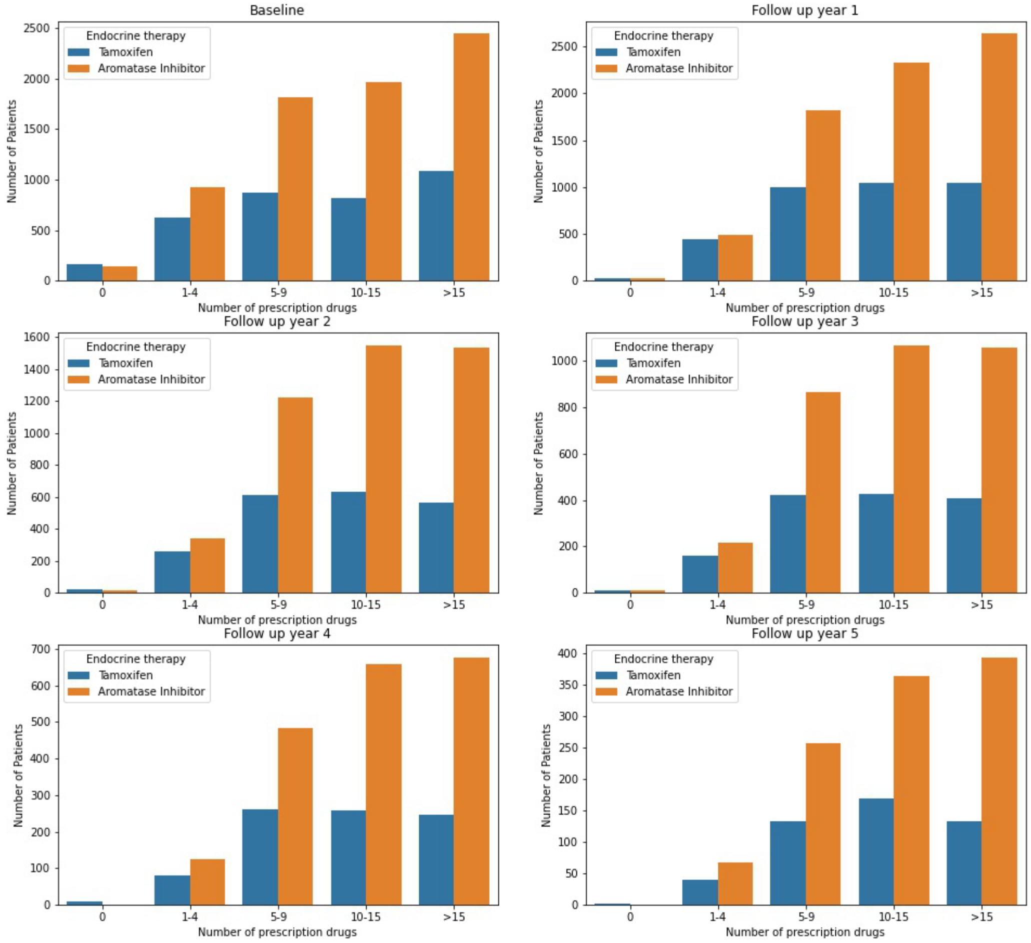

© 2022 Rassy E et al. *JAMA Network Open.*

eFigure 2. Proportion of Patients Receiving Treatments That Manage Adverse Events Potentially Caused by Endocrine Therapy

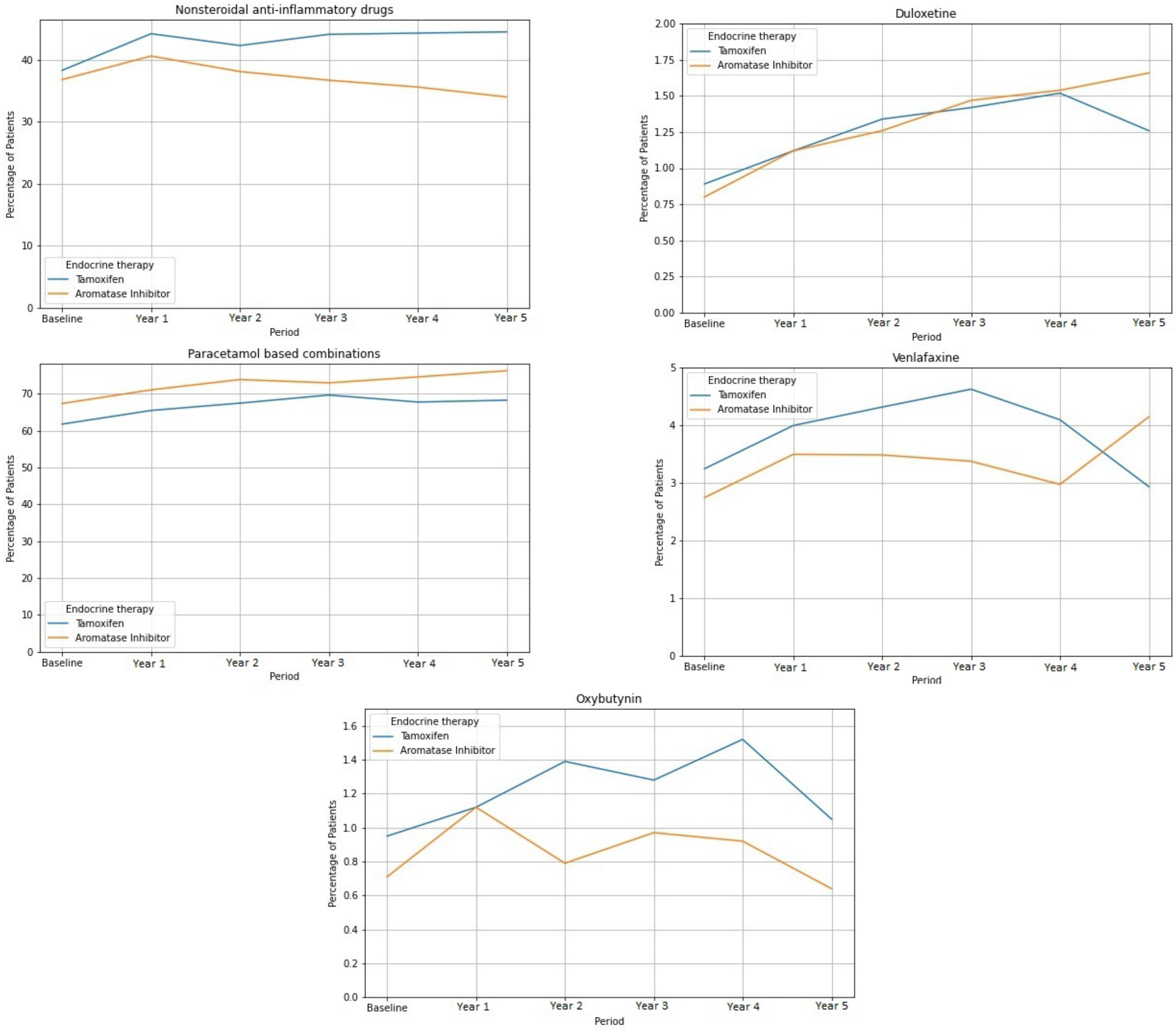

© 2022 Rassy E et al. *JAMA Network Open.*

eFigure 3. Sensitivity Analyses of the Multivariable Models of Adherence Adjusted for Age, Comedication, Adherence During the Previous Year, With and Without PDDI (Model 1 and 2, Respectively)

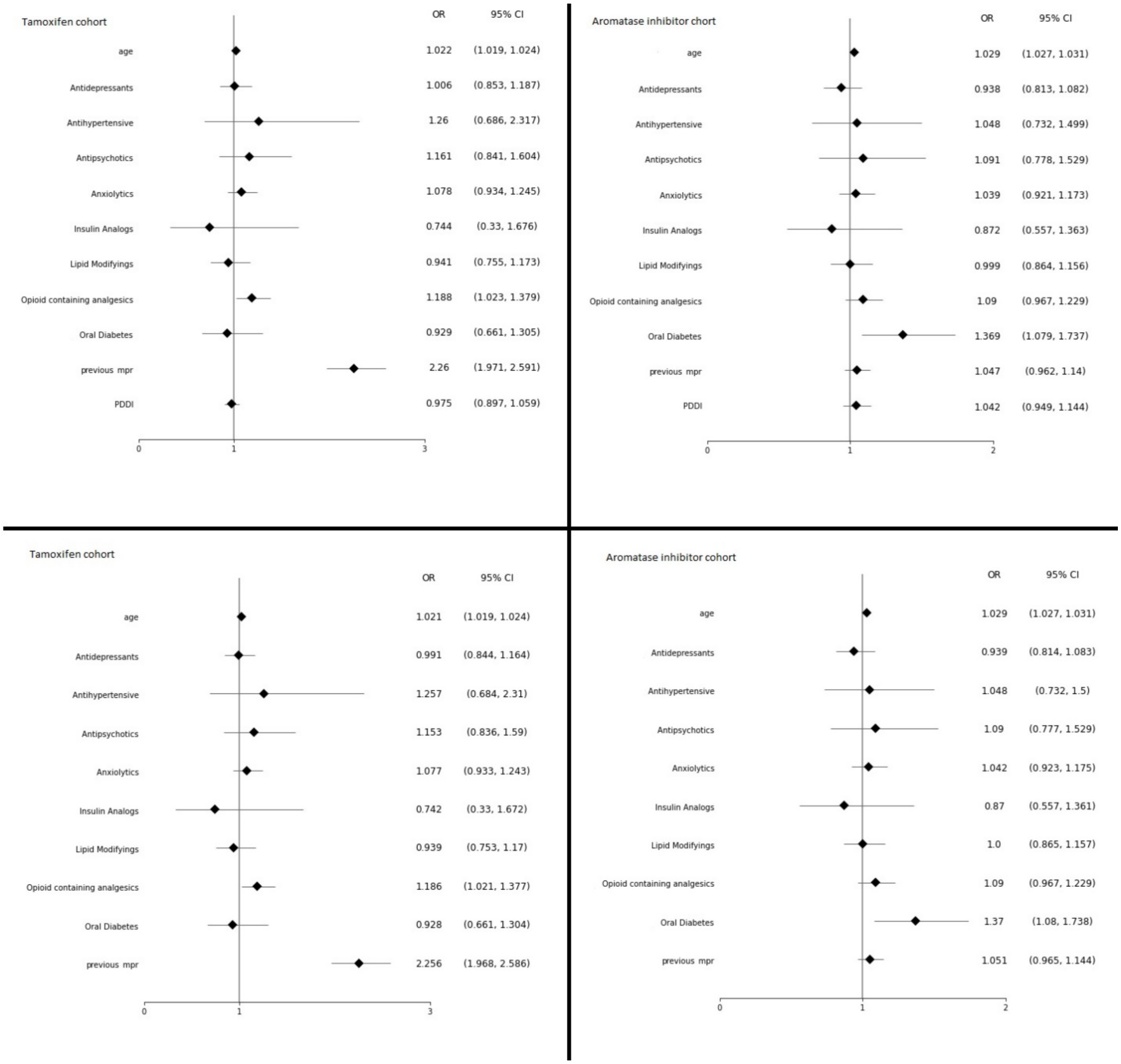

© 2022 Rassy E et al. *JAMA Network Open.*
